# Applying quality improvement methods to neglected conditions: development of the South Asia Burn Registry (SABR)

**DOI:** 10.1186/s13104-019-4063-0

**Published:** 2019-01-29

**Authors:** Nukhba Zia, Asad Latif, Saidur Rahman Mashreky, Ehmer Al-Ibran, Madiha Hashmi, A. K. M. Fazlur Rahman, Sazzad Khondoker, Mohammed Saeed Quraishy, Adnan A. Hyder

**Affiliations:** 1 Johns Hopkins International Injury Research Unit, Department of International Health, Johns Hopkins Bloomberg School of Public Health, Baltimore, MD 21205 USA; 2 Department of Anesthesiology and Critical Care Medicine, Johns Hopkins University School of Medicine, Baltimore, MD 21205 USA; 3 Centre for Injury Prevention Research, Dhaka, 1206 Bangladesh; 4 Burns Centre, Civil Hospital, Karachi, 74200 Pakistan; 5 Department of Anaesthesia, Aga Khan University, Karachi, 74800 Pakistan; 6 National Institute of Burn and Plastic Surgery, Dhaka, Bangladesh; 7 Civil Hospital, Karachi, 74200 Pakistan; 8 Milken Institute School of Public Health, George Washington University, Washington, DC USA

**Keywords:** Burn injuries, Burn care, Registry, Quality of care, Resource-constraint settings, Bangladesh, Pakistan, South Asia

## Abstract

**Objective:**

South Asia has the highest mortality rate from burns in the world. Application of quality improvement methods to burn care can help identify health system gaps. Our overall aim is to introduce a sustainable hospital-based burn registry for resource-constrained settings to assess health outcomes of burn injury patients presenting to dedicated burn injury centers in South Asia.

**Results:**

The South Asia Burn Registry (SABR) is implemented through collaborative approach in selected burn centers in Bangladesh and Pakistan. Th registry collects data on burn injury events, the care provided, and the functional status of patients at discharge from burn centers. It covers the entire spectrum of care provision for burn injury patients from the actual event through their discharge from the healthcare system. SABR investigates locally relevant contextual factors associated with burn injury and health-system requirements for burn patients receiving emergency and inpatient care in resource-constrained settings. It also explores factors associated with burn injury and care provision. SABR will inform better prevention and management efforts in South Asia and help to address healthcare needs of burn injury patients.

**Electronic supplementary material:**

The online version of this article (10.1186/s13104-019-4063-0) contains supplementary material, which is available to authorized users.

## Introduction

Burn injuries have devastating sequelae including both physical impairment and psychosocial consequences [1–3]. Each year, almost 11 million people seek medical attention for severe burn injury, and over 265,000 die from burns worldwide [4]. These numbers are probably an underestimation of the actual burden, as many patients do not seek medical attention, especially in low- and middle-income countries (LMICs) [1, 5]. The 2017 Global Burden of Disease estimated the global burn mortality rate to be 1.6 per 100,000 population [6]. South Asia region accounts for half of these deaths with annual incidence for burns estimated to be as high as 187–243 per 100,000 [7].

In South Asia extremes of age predisposes people to burn injuries; children under 5 years seem to be at particular risk, with nearly half of all childhood burns occurring in male infants [8]. Older women in the region are also particularly vulnerable to fire-related injuries. Gender discrepancies are reported between South Asian countries, with females accounting for a larger proportion of burns in India and Pakistan, but not in Bangladesh [9–12]. Regional data suggests local clothing customs, domestic violence, and self-harm are important risk factors associated with burn injuries [13–16].

Disease-specific databases such as trauma and cancer registries have been found to be useful at national or regional levels [17–19]. The World Health Organization (WHO) has developed a global burn registry to estimate burn injuries and associated risk factors [20]. The National Burn Repository in the United States [21] and the Bi-National Burn Registry in Australia and New Zealand [22] are examples of successfully implemented burn registries in high-income countries (HICs). In LMICs, development of such registries based on available resources is necessary to gain an understanding of the local context and burn care processes to develop future interventions [23]. Registries assess risk factors associated with injury and help agencies to set priorities for resource allocation that can support burn care in LMICs through longitudinal data collection [24].

Current work doesn’t address the need for assessing quality of burn care which results in lack of developing and implementing measures that can help to improve standard of care. In the context of burns, infection control, fluid management, wound care are critical for patient outcome and survival [1, 17, 25]. Such limitations mean that these studies often do not capture potential gaps in the existing burn-related health care system. This study addresses this gap by proposing South Asia Burn Registry (SABR)—a paper-based registry that is piloted as a data-driven quality improvement tool for resource-constraint settings. The overall aim is to propose a sustainable burn registry for LMICs and describe an approach taken to determine its feasibility, acceptability, and utility.

## Main text

Merely having a data system is not enough; it is crucial to apply the data to a framework that helps to improve quality of care with standard clinical guidelines and eventually patient outcomes [26]. The Institute for Healthcare Improvement (IHI) framework is a model that focuses on identifying improvement in care and guides the process through a Plan-Do-Study-Act (PDSA) cycle [27]. With this approach, specific measures to assess improvement within a system can be established, and tested through reiterations of the PDSA cycle to provide real-time data on whether the change resulted in actual improvement [27].

The four stages of PDSA are; “Planning” identifies aspect of care that needs change and plan is developed to address the change, “Do” tests the identified change through execution of the plan and allows for observations to be made to document the problem associated with the plan, “Study” analyses data, and identifies successes and failures of the change and “Act” addresses additional changes that need to be made in the current system, allows adaptations to be made and implemented for the next round of PDSA (Table 1) [28].

**Table 1:** Application of PDSA cycle to South Asia Burn Registry [26]

PDSA step	Description	SABR contribution
Plan	Identify an aspect of a system that needs change and develop a plan to address the change	Use of regular data collection (patient demographics, burn injury details, care provided and outcomes) by health care providers to assess current care plan and identify and devise plan for areas of care that need change
Do	Test the identified change through execution of the plan and allow documentation of the problem(s) associated with the plan	Monitor and evaluate changes in care plan by using data to identify benefits/harms from adaptations in care plan
Study	Analyze and synthesize the data and highlight the success and failures of the change being studied	Develop indicators to quantify the change and identify pitfalls in the plan Potential for synthesis of indicators into assessment of quality of burn care
Act	Address further changes that need to be made in the current system and allow adaptations related to the change and implement them for next round of PDSA cycle	Develop automated reporting of validated indicators and generation of reports of locally identified processes and parameters of interest Help to contextualize local burn care and adherence to best practices

SABR serves as a key component of a quality improvement framework for healthcare providers (physicians, surgeons, nurses, and technicians, as well as rehabilitation and occupational services) and managers to improve the gaps present in burn care at the selected sites. The PDSA framework considers a multifaceted approach that is crucial for the utilization of data being collected through implementation of SABR (Table 1). This framework is critical for successful deployment and utilization of the SABR registry through the following mechanisms [29].

### Iterative process

The use of SABR as part of a PDSA tool will facilitate planning and implementation of system-level changes that are data-driven. E.g., the risk of mortality is high for individuals with inhalational burns, SABR can help to identify care-related factors that will facilitate early identification and management of such burns. These changes can be implemented into the current practice and tested using the PDSA method to improve burn outcomes.

### Prediction-based test of change

Through PDSA framework, SABR data will predict patient outcomes based on the changes implemented in the system. E.g. indicators like extremes of age, inhalational burns, and third-degree burns are predictors of mortality; however, identification of these indicators and rigorous management improves patient outcomes [1]. Data on these indicators will help care-providers to predict patient outcomes before and after implementation of an intervention (early initiation of treatment) at the burn centre [29].

### Small-scale testing

The feedback loop, inherent to PDSA, is crucial to the success of SABR, as it will enable almost real-time generation of evidence related to a change in patient management through early treatment initiation. Care-providers can plan the timeframe during which changes are tested to determine the results.

### Data use over time

SABR will be integrated into the current burn-care system in Bangladesh and Pakistan. It will allow observation of the variations in burn types and associated care over a period of time [29]. Such variation helps registry users to better understand the system and facilitate planning of evaluations related to outcome and impact of an intervention.

### Documentation

SABR will generate data to support the changes in care made and tested in the system with the PDSA approach [29]. The documentation of each step of the PDSA cycle will provide insight into the local settings and their challenges related to burn care (Table 1).

SABR is a collaborative effort between the Johns Hopkins International Injury Research Unit hosted by the Department of International Health, Johns Hopkins Bloomberg School of Public Health, USA (JH-IIRU); the Centre for Injury Prevention and Research, Bangladesh (CIPRB); the National Institute of Burn and Plastic Surgery, Bangladesh (NIBPS); Aga Khan University, Pakistan (AKU); and Civil Hospital Karachi, Pakistan (CHK) (Additional file 1: Table S1). NIBPS and CHK are dedicated burn centres that cater to burn injuries in their respective cities and worked closely with CIPRB and AKU during SABR pilot.

The participating institutes have a long-standing history of collaboration in injury and trauma research in the region. This includes development and implementation of national and community-level injury surveillance systems, childhood injuries, drowning and trauma registry development [30–33]. SABR builds on this with the intention of bringing a data-based approach to assess, and ultimately improve, the type and quality of care for burn injuries. In addition, there is engagement with administration and care-providers right from the conception of this work which has led to the development of draft SABR tool.

As part of the initial work, SABR tool was developed based on literature review; burn registries from HICs; and expert opinion from researchers and physicians working in the field of burn injuries [1, 5, 10, 12, 13, 17, 21, 22, 34, 35]. The current draft of SABR data collection tool comprises five sections (Table 2). Section 1 focuses on initial assessment of burn patients in the emergency department of burn centres. This information is collected through patient observation. Section 2 relates to patient demographics collected through interview of the patient or next of kin. Section 3 records information related to the burn injury event through interview of the patient or next of kin and from medical records. Sections 4 and 5 focuses on patients admitted to a burn centre for further care and covers the hospital course of these patients. Section 4 is completed at the time of patient discharge through interview of the patient or next of kin and a review of the patient’s medical record. Section 5 covers information related to laboratory tests done during the hospital stay and is recorded from the existing patient medical record (Table 2).

**Table 2:** Description of the South Asia Burn Registry (SABR) tool

Section	Description and example variables
1: Initial assessment information—ED	The information for this section is collected through patient observation in the ED. It includes date and time of injury, date and time of presentation to the burn center, referral cases, first aid received or not, any treatment received prior to burn center arrival
2: Patient demographics	The data for this section is collected from patient/next of kin interviews and includes patient’s age, sex, area of residence, marital status, education level, occupation, household members
3: Burn injury information	The data for this section is collected from patient/next of kin interviews and ED medical records. Data includes place of burn injury; activity; type and cause of burn; injury event; comorbid conditions; body region and percentage area burned; intent; predisposing factors like alcohol use, cigarette smoking, drugs, and physical disability; ED management and disposition
4: Hospital course	The information for this section is collected through interview of patient/next of kin at the time of burn center discharge and from hospital medical records. Data includes requirement of nutritional support, blood products, dialysis, antibiotics, in-patient complications, number and type of surgeries, patient disposition, and functional assessment at the time of disposition. Details related to ICU stay such as duration of mechanical ventilation are also collected
5: Clinical parameters	Data is collected from hospital medical records and includes weight, height, hemoglobin, platelets, electrolytes, renal function, and urine output

Full tool is available as a Additional file 2

*ED* emergency department, *ICU* intensive care unit

**Fig. 1:**
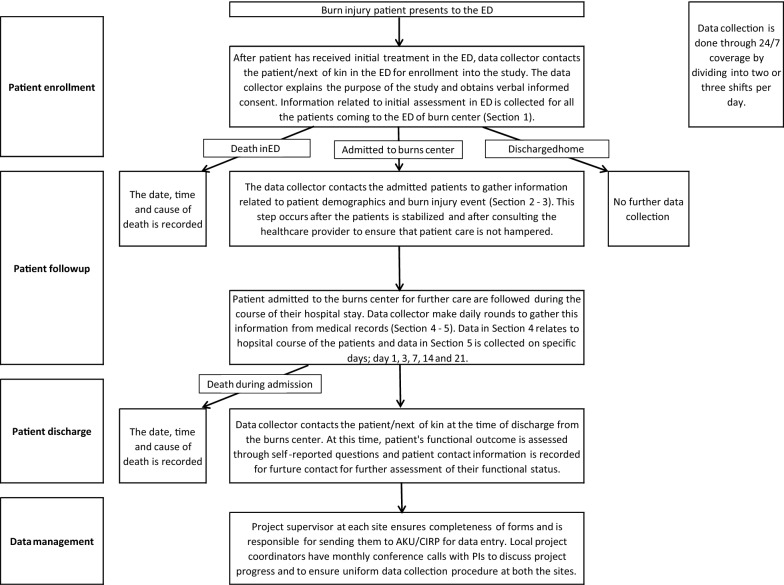
Work flow for The South Asia Burn Registry (SABR) project

SABR is using the existing healthcare infrastructure and resources. In this regard, administration staff and care-providers at the centres were trained. However, since this a pilot, dedicated data collection team is also hired and trained at each site to help the current staff and care-providers at the burn centres during the pilot. This model will help in transition of SABR from its pilot phase to its full-fledged implementation in the current systems of burn care. SABR collects data on all burn injury patients presenting to the selected burn centres for the first time. All adult and paediatric, male and female patients are enrolled after receiving oral consent and assent from adults and children with burn injury (Fig. 1). The first contact is at the time of admission to determine the circumstance in which the burn injury occurred; the second contact, at the time of discharge, is used to assess level of disability and outcome and determine the experience of patients or next of kin in terms of hospital stay and care provided.

## Limitations

This multi-site study is looking to investigate both event and health system related data on burn patients receiving inpatient care in LMICs. We are hopeful that this process will empower these burn centres to evaluate the quality of their services for burn patients in the local context [36, 37]. Thus, SABR will have a dual role of *data generation* and *data utilization* for quality improvement and care practices related to burn care in settings like Bangladesh and Pakistan. Some limitations of SABR include: *First,* this work will identify issues around quality of burn care in LMICs. This may not be seen in positive light as many burn centres function within limited resources. However, due to PDSA cycle inherent within SABR implementation, this registry has the potential for use as a quality improvement tool. It will be able to generate evidence that can guide the administration of the burn centres to budget for its sustainability once the pilot phase is completed.

*Second*, SABR is the first attempt at implementing a facility based-burns registry in the region, thus, there is no benchmark to compare its results. However, findings from the study will provide a validated, locally relevant tool to serve as the basis of future regional burn registries.

*Third*, engaging with stakeholders and their continuous commitment to use SABR as a data collection tool for improving quality of burn care is crucial. Having a dedicated trained administration staff for overall oversight and management of SABR will also be a challenge. Stakeholder engagement in this regard is very crucial and therefore in the pilot phase of implementation administration staff and care-providers will be engaged so that they understand its utilization and application in burn care. This may result in some unease and providers may consider it a burden, however, having trained administration staff will help to reduce burden on care-providers.

*Fourth*, integration of SABR into the current system is another anticipated challenge because these burn centres have been providing burn care for many decades. Change in their practices based on data and evidence may be difficult, however, the current interest of the stakeholders and their willingness to implement SABR in the burn centres is a testament to their drive for bringing change in the current burn care and management practices. Stakeholders and collaborators work together to address issues around feasibility of registry use and modify SABR after the pilot phase and before it is available for scale-up.

SABR is a data system that is developed and implemented for resource-constraint settings like South Asia. It leverages existing healthcare systems in Dhaka, Bangladesh and Karachi, Pakistan to generate evidence to address gaps in provision of burn care in resource-constrained settings.

## Additional files

**Additional file 1: Table S1.** Participating organizations.

**Additional file 2.** SABR tool.

## Abbreviations

AKU: Aga Khan University
CHK: Civil Hospital Karachi
CIPRB: Centre for Injury Prevention and Research, Bangladesh
HICs: high-income countries
JH-IIRU: Johns Hopkins International Injury Research Unit
LMICs: low- and middle-income countries
NIBPS: National Institute of Burn and Plastic Surgery
PDSA: Plan-Do-Study-Act cycle
SABR: South Asia Burn Registry
WHO: World Health Organization
