# Supplementary material for: Applying quality improvement methods to neglected conditions: development of the South Asia Burn Registry (SABR)
Source: BMC Res Notes. 2019 Jan 29;12:64. doi: 10.1186/s13104-019-4063-0 (PMC6352446; doi:10.1186/s13104-019-4063-0)
Supplement: Supplementary file 1 — Additional file 1: Table S1. Participating organizations. [file 13104_2019_4063_MOESM1_ESM.pdf]

Supplementary Table 1. Participating organizations

Organization	Type	Location	Primary role
JH-IIRU	Academic center	Baltimore, MD, USA	Technical support, ethics- and data-related training
AKU	Academic university	Karachi, Pakistan	Study coordination across the sites, training of data collection teams, data management and supervision, communication with JH-IIRU
CHK	Academic university-affiliated hospital	Karachi, Pakistan	Data collection supervision, coordination of activities with AKU
CIPRB	Research center	Dhaka, Bangladesh	Study coordination with AKU, training of data collection teams, data management and supervision
NIBPS	Academic university-affiliated hospital	Dhaka, Bangladesh	Data collection supervision, coordination of activities with AKU and CIPRB

AKU, Aga Khan University; CHK, Civil Hospital Karachi; CIPRB, Centre for Injury Prevention and Research; NIBPS, National Institute of Burn and Plastic Surgery; JH-IIRU, Johns Hopkins International Injury Research Unit.
