# Supplementary material for: Applying quality improvement methods to neglected conditions: development of the South Asia Burn Registry (SABR)
Source: BMC Res Notes. 2019 Jan 29;12:64. doi: 10.1186/s13104-019-4063-0 (PMC6352446; doi:10.1186/s13104-019-4063-0)
Supplement: Supplementary file 2 — Additional file 2. SABR tool. [file 13104_2019_4063_MOESM2_ESM.pdf]

The South Asia Burn Registry (SABR) Project

Case ID ______________________ **Center ID** ______________________

Sections 1 - 3 to be completed at the time of presentation to the emergency department of the burn center.

New patient ☐ **Admitted patient** ☐

**Section 1: INITIAL ASSESSMENT INFORMATION – ED** *(The information in this section will be collected through observation in the ED)*			
**Date of injury (dd/mm/year)**		**Time of injury (hh:mm)**	
**Date of presentation (dd/mm/year)**		**Time at presentation (hh:mm)**	
**Date of initial ED assessment (dd/mm/year)**		**Time at initial ED assessment (hh:mm)**	
**Date of initial ED treatment (dd/mm/year)**		**Time at initial ED treatment (hh:mm)**	
**Referred case (from another facility)**	1. Yes	2. No	98. Unknown
**Referral source (if referred case)**	1. Self	2. Hospital	3. Private clinic
	98. Unknown	99. Other (specify)	
**Receive treatment before coming to the burn center**	1. Yes	2. No	98. Unknown
**If yes, what type of treatment**			
**Arrival method**	1. Private vehicle	2. Taxi/rickshaw	3. Ambulance
	4. Police	98. Unknown	
	99. Other (specify)		
**Level of training of person who did initial assessment in ED**	1. Intern/ House officer	2. Resident/ postgraduate trainee	3. Chief resident
	4. Consultant	5. Non-physician healthcare provider	98. Unknown
	99. Other (specify)		
**Availability of surgeon at the time of patient presentation**	1. Yes	2. No	98. Unknown

© Johns Hopkins International Injury Research Unit

**ED interventions* (multiple response)**	1. Oxygen	2. Fluids (crystalloids)	3. Fluids (colloids)
	4. Fluids (blood products)	5. Intubation	6. Systemic antibiotics
	7. Topical antibiotics	8. Topical agents (e.g. Silver Sulfadiazine)	9. Cooling
	10. Wound debridement	11. Wound dressing	98. Unknown
	99. Other (specify)		
**Tetanus prophylaxis ***	1. Yes	2. No	98. Unknown
**Disposition from ED***	1. Discharged home	2. Discharged to another facility	3. Admitted to ICU
	4. Admitted to ward	5. Taken to operating room	6. Left against medical advice
	7. Death	98. Unknown	
	99. Other (specify)		
**If case of death in ED**			
**Date of death (dd/mm/year)**		**Time of death (hh:mm)**	
**Cause of death**	1. Cardiac arrest	2. Respiratory failure	3. Renal failure
	4. Sepsis	5. Preexisting condition	6. Shock (hypovolemic)
	98. Unknown	99. Other (specify)	

© Johns Hopkins International Injury Research Unit
October 2013

Case ID ______________________ **Center ID** ______________________

Section 2: PATIENT DEMOGRAPHIC INFORMATION *(The information for this section will be collected through interview of patient/next of kin)*			
**Age (mths/yrs)**		**Date of birth (dd/mm/yy)**	
**Gender**	1. Male	2. Female	98. Unknown
**Area of residence**	1. Urban	2. Rural	98. Unknown
**Location of residence**			
**Marital status of burn injury patient**	1. Single	2. Married	3. Divorced
	4. Widowed	98. Unknown	
**Education level of the burn injury patient**	1. None	2. Informal	3. Up to class 5
	4. Up to class 8	5. Up to class 10	6. Up to class 12
	7. Bachelors	8. Masters	9. Diploma
	10. Professional (MBBS, MBA etc)		98. Unknown
	99.Other (specify)		
**Occupation of the burn injury patient**	1. Housewife	2. Unemployed	3. Manual laborer
	4. Agriculture	5. Industrial	6. Maids/cooks
	7. Retired	8. Student	98. Unknown
	99. Other (specify)		
**Monthly income**	1. <10,000	2. 10,000 – 30,000	3. 30,000 – 50,000
	4. >50,000	98. Unknown	
	99. Other (specify)		
**In case patient is a child ≤ 17 years complete the following**			
**Respondent**	1. Father	2. Mother	3. Grandparent
	4. Aunt/Uncle	5. Sibling	98. Unknown
	99. Other(specify)		
**Does the child goes to school**	1. Yes	2. No	98. Unknown
**If yes, which grade/class**			
**Occupation of father**	1. Unemployed	2. Manual laborer	3. Agriculture
	4. Industrial	5. Retired	6. Private job
	98. Unknown	99. Other (specify)	

© Johns Hopkins International Injury Research Unit
October 2013

**Occupation of mother**	1. Housewife	2. Maids/cooks	3. Office job
	4. Retired	98. Unknown	
	99. Other (specify)		
**Education level of father**	1. None	2. Informal	3. Up to class 5
	4. Up to class 8	5. Up to class 10	6. Up to class 12
	7. Bachelors	8. Masters	9. Diploma
	10. Professional (MBBS, MBA etc)		98. Unknown
	99. Other (specify)		
**Education level of mother**	1. None	2. Informal	3. Up to class 5
	4. Up to class 8	5. Up to class 10	6. Up to class 12
	7. Bachelors	8. Masters	9. Diploma
	10. Professional (MBBS, MBA etc)		98. Unknown
	99. Other (specify)		

© Johns Hopkins International Injury Research Unit

Case ID ______________________ **Center ID** ______________________

**Section 3: BURN INJURY INFORMATION** *(The information for this section will be collected through interview of patient/next of kin and review of ED medical record*)*			
**Physical location at the time of burn injury**	Town ______________________	City ______________________	
**Place burn injury occurred**	1. Kitchen	2. Bathroom	3. Other areas of home
	4. School	5. Street/road	6. Sports area
	7. Industrial area	8. Commercial area	9. Recreational area
	10. Construction area	11. Farm	12. Countryside
	13. Medical service area	14. Residential homes	98. Unknown
	99. Other (specify)		
**Activity when burn injury occurred**	1. Cooking	2. Work (home)	3.Work (outside home)
	4. Bathing	5. Travelling	6. Sports
	7. Education	8. Agricultural activity	9. Industrial
	98. Unknown	99. Other (specify)	
**Type of burn**	1. Flash	2. Flame/fire	3. Scald
	4. Contact	5. Cooling	6. Non-flame burns
	7. Chemical	8. Electrical	9. Radiation
	98. Unknown	99. Other (specify)	
**Cause of burn (including fuels)**	1. Natural gas	2. Gasoline	3. Other flammable liquids
	4. Hot liquid (water, oil, grease, tea)	5. Kerosene oil	6. Plastics, glass, hot metals
	7. Liquefied petroleum gas (LPG)	8. Coal	9. Biofuel eg cow dung
	10. Wood	11. Acid	12. Alkali
	13. High voltage wires	14. Low voltage (power outlet/home appliances	15. Lightning
	16. Spirit (ethanol or alcohol)	98. Unknown	
	99. Other (specify)		
**Source of fuel**	1. Stove	2. Lantern	3. Generator
	4. Match sticks	5. Water tanks	6. Liquefied petroleum gas (LPG) cylinders
	7. Contact	98. Unknown	
	99. Other (specify)		

© Johns Hopkins International Injury Research Unit

**Injury event**	1. Stove accident	2. Cylinder explosion	3. Gas collected in room, water tank
	4. Clothes catching fire	5. Fuel spill	6. Epileptic fit
	98. Unknown	99. Other (specify)	
**Suspected intent**	1. Unintentional	2. Suicide/attempted suicide	3. Assault
	98. Unknown	99. Other (specify)	
**Clinical suspicion of alcohol use***	1. Yes	2. No	98. Unknown
**Clinical suspicion of use of drugs***	1. Yes	2. No	98. Unknown
**Hx of smoking**	1. Yes	2. No	98. Unknown
**Any physical disability before burn injury**	1. Yes	2. No	98. Unknown
**Any mental disability before burn injury**	1. Yes	2. No	98. Unknown
**First aid given prior to presentation**	1. Yes	2. No	98. Unknown
**First aid description (if available)**			
**Comorbid conditions (multiple response)**	1. None	2. HTN	3. CVD
	4. Arrhythmias	5. Stroke	6. Seizure disorder
	7. Dementia	8. Psychiatric illness	9. Diabetes
	10. Lung disease	11. Renal disease	98. Unknown
	99.Other (specify)		
**Anatomic burn location in % of total BSA * (multiple response)**	1. Head	2. Face	3. Upper limbs
	4. Hands	5. Chest	6. Abdomen
	7. Back	8. Lower limbs	9. Feet
	10. Perineum	98. Unknown	
	99. Other (specify)		
**Associated injuries* (Multiple response)**	1. None	2. Polytrauma	3. Head injury (open)
	4. Head injury (closed)	5. Spinal cord injury	6. Neurological injury
	7. Ocular injury	8. Ear injury	9. Thoracic/chest injury
	10. Abdominal injury	11. Pelvic fracture	12. Long bone fracture
	98. Unknown	99. Other (specify)	

© Johns Hopkins International Injury Research Unit

**% total burn size***<br>**(2nd & 3rd degree burns)**			
**Evidence of inhalation injury at time of presentation***			
a. Mental confusion, unconsciousness	1. Yes	2. No	98. Unknown
b. Facial burns	1. Yes	2. No	98. Unknown
3. Singing of facial hair	1. Yes	2. No	98. Unknown
3. Soot in mouth, around nares or in sputum	1. Yes	2. No	98. Unknown
3. Hoarseness, stridor	1. Yes	2. No	98. Unknown
**ED interventions***<br>**(multiple response)**	1. Oxygen	2. Fluids (crystalloids)	3. Fluids (colloids)
	4. Fluids (blood products)	5. Intubation	6. Systemic antibiotics
	7. Topical antibiotics	8. Topical agents<br>(e.g. Silver Sulfadiazine)	9. Cooling
	10. Wound debridement	11. Wound dressing	98. Unknown
	99. Others(specify)		
**Tetanus prophylaxis ***	1. Yes	2. No	98. Unknown
**Disposition from ED***	1. Discharged home	2. Discharged to another facility	3. Admitted to ICU
	4. Admitted to ward	5. Operating room	6. Left against medical advice
	7. Death	98. Unknown	
	99. Other (specify)		
**If case of death in ED***			
**Date of death**<br>**(dd/mm/year)**		**Time of death**<br>**(hh:mm)**	
**Cause of death**	1. Cardiac arrest	2. Respiratory failure	3. Renal failure
	4. Sepsis	5. Preexisting condition	6. Shock (hypovolemic)
	98. Unknown	99. Other (specify)	

© Johns Hopkins International Injury Research Unit

Section 4 and 5 needs to be completed at the time of discharge from the burn center

Section 4: HOSPITAL COURSE

(The information for this section will be collected through interview of patient/next of kin at the time of discharge from the burn center and hospital medical record*)

**Discharge diagnosis* (top three)**			
**Complications during hospital stay* (multiple response)**	1. None	2. Neurological	3. Pulmonary
	4. Cardiovascular	5. Abdominal	6. Metabolic
	7. Musculoskeletal	8. Sepsis/infection	9. Psychiatric
	98. Unknown	99. Other (specify)	
**Evidence of inhalation injury during hospital stay***			
a. Mental confusion, unconsciousness	1. Yes	2. No	98. Unknown
b. Facial burns	1. Yes	2. No	98. Unknown
c. Singing of facial hair	1. Yes	2. No	98. Unknown
d. Soot in mouth, around nares or in sputum	1. Yes	2. No	98. Unknown
e. Hoarseness, stridor	1. Yes	2. No	98. Unknown
**Dialysis***	1. Yes	2. No	98. Unknown
**Blood products***	1. Yes	2. No	98. Unknown
**Quantity of blood products***			
**Nutritional support***	1. Yes	2. No	98. Unknown
**Antibiotics ***	1. Yes	2. No	98. Unknown
**Duration of antibiotics***	(days)		
**Albumin***	1. Yes	2. No	98. Unknown
**Immunoglobulins***	1. Yes	2. No	98. Unknown
**ICU admission after admission to the hospital ***	1. Yes	2. No	98. Unknown
**If admitted to ICU***			
**Mechanical ventilation**	1. Yes	2. No	98. Unknown
**Mechanical ventilation duration**	(days)		
**Inotropic support**	1. Yes	2. No	98. Unknown
**Duration of inotropic support**	(days)		
**Bronchoscopy**	1. Yes	2. No	98. Unknown

© Johns Hopkins International Injury Research Unit

**Central line placement**	1. Yes	2. No	98. Unknown
**Duration of central line usage**	(days)		
**Dialysis**	1. Yes	2. No	98. Unknown
**ICU complications (if any)**	1. Pneumonia	2. Respiratory failure	3. Renal failure
	4. Sepsis	5. Line infection	98. Unknown
	99. Other (specify)		
**Duration of ICU stay**	(days)		

Surgical procedures*

**Surgery**	1. Yes	2. No	98. Unknown
**Number of surgeries**			
**Type of surgeries (multiple response)**	1. Incision and drainage	2. Debridement	3. Escharotomy
	4. Split Skin Grafting	98. Unknown	
	99.Other (specify)		
**Split Skin Grafting**	1. <48 hours	2. <1 week	3. ≥1 week
**Number of Split Skin Grafting**			
**Duration of hospital stay**	(days)		
**Disposition from hospital**	1. Discharged home	2. Discharged to another hospital	3. Left against medical advice
	4. Rehabilitation	5. Death	98. Unknown
	99. Other (specify)		
**Payment**	1. Employer	2. Insurance	3. Charity
	98. Unknown	99. Other (specify)	

Functional assessment at discharge from the hospital

**How much difficulty in:**	**None**	**Mild**	**Moderate**	**Severe**	**Extreme or cannot do**	**With assistance**
**Standing**						
**Walking**						
**Eating**						
**Going to bathroom**						
**Getting dressed**						

In case of death during hospital stay*

**Date of death (dd/mm/year)**		**Time of death (hh:mm)**	
**Cause of death**	1. Pneumonia	2. Respiratory failure	3. Renal failure
	4. Sepsis	5. Preexisting condition	98. Unknown
	99. Other (specify)		

© Johns Hopkins International Injury Research Unit
October 2013

Case ID ______________________ **Center ID** ______________________

Section 5: CLINICAL PARAMETERS

(The information for this section will be collected from hospital medical record)

	Day 1	Day 3	Day 7	Day 14	Day 21
Weight (in kg)					
Height					
Hemoglobin					
WBC					
Platelets					
Na					
K					
Cl					
HCO_3					
Creatinine					
BUN					
Albumin					
Cultures					
Blood					
Urine					
Wound					
Others					
24 hr urine output					
ABGs					
pH					
pO_2					
pCO_2					
HCO_3					

Form completed by (Name & signature) ____

Date: ______________

© Johns Hopkins International Injury Research Unit
October 2013
